# The effect of nerve growth factor on differentiation of corneal limbal epithelial cells to conjunctival goblet cells in vitro

**Published:** 2010-12-15

**Authors:** Weiwei Li, Xuguang Sun, Zhiqun Wang, Ran Li, Li Li

**Affiliations:** Beijing Institute of Ophthalmology, Beijing Ophthalmology & Visual Sciences Key Lab, Beijing Tongren Eye Center, Beijing Tongren Hospital, Capital Medical University, Bejing, China

## Abstract

**Purpose:**

To evaluate in vitro the effect of nerve growth factor (NGF) on the differentiation of mouse corneal limbal progenitor cells into goblet cells and to observe the expression of mucin-5AC (*MUC5AC*) mRNA.

**Methods:**

Mouse limbal epithelial cells were cultured in a 1:1 mixture of Dulbecco’s modified Eagle’s medium/Ham’s nutrient mixture F12 in vitro, and 63-kDa protein (p63) in cultured cells was identified with immunofluorescence staining. Different groups of the cultured cells were exposed to NGF at different concentrations (0 ng/ml, 10 ng/ml, 100 ng/ml, and 250 ng/ml). *MUC5AC* gene expression (real-time PCR) and goblet cell differentiation (MUC5AC immunofluorescence staining) were analyzed at different time points (24 h, 72 h, and 5 d).

**Results:**

In primary culture, the limbal epithelial cells were compact, uniform, and cobblestone pavement in shape. Some limbal epithelial cells were positive for p63. The MUC5AC-positive cells were detected when the cells were treated with 100 ng/ml NGF at each time point and with 250 ng/ml NGF at 5 d. The expression of *MUC5AC* mRNA increased when using 100 ng/ml NGF. The MUC5AC-positive cells were not detected when 0 ng/ml and 10 ng/ml NGF were used at each time point.

**Conclusions:**

The results of this study suggest that NGF might promote the differentiation of corneal limbal progenitor cells into conjunctival goblet cells and upregulate the expression of *MUC5AC* mRNA in primary culture. Further studies using an animal model in vivo are needed.

## Introduction

Mucins of the ocular surface are very important components of the tear film. They play a critical role in the protection of the corneal and conjunctival epithelium. Mucin-5AC (MUC5AC) is the most prevalent secreted mucin produced by conjunctival goblet cells. The functions of secreted mucins include lubrication, clearance of allergens and pathogens, and antimicrobial activity [1].

Some ocular surface diseases are significantly associated with the decrease of mucins and a shortage of goblet cells, such as dry eye syndromes, long-term contact lens wear [2], and ocular allergies [3]. As a result, the stimulation of mucin secretion pathways becomes a new strategy for the treatment of mucin-related ocular surface diseases. From this point of view, increasing the number of goblet cells is a potential therapy for increasing mucin secretion.

It has been shown that goblet cells and conjunctival epithelial cells come from conjunctival progenitor cells and later transient amplifying cells [4]. Some evidence indicates that corneal limbal progenitor cells have the capacity to generate goblet cells under conjunctival wound conditions, suggested that corneal limbal progenitor cells have “oligopotence” [5]. It has been demonstrated that several growth factors were able to modulate the proliferation and differentiation of corneal limbal epithelial cells [6]. Nerve Growth Factor (NGF) is one of these kinds of factors. Recent studies demonstrated that NGF not only can improve the proliferation and differentiation of corneal limbal epithelial cells in vitro [6], but also can promote corneal healing after injury in vivo [7]. Meanwhile, studies of conjunctival goblet cells have shown that NGF can stimulate mucin secretion [8], topical application of NGF can increase the number of goblet cells in dogs affected by surgical dry eye [9], and NGF receptors can be expressed by conjunctival and corneal epithelial cells [10]. NGF may be closely related to ocular surface mucins.

In this study, the effect of NGF on the differentiation of corneal limbal progenitor cells into conjunctival goblet cells was evaluated, and *MUC5AC* expression in primary epithelial cultures was measured.

## Methods

### Animals

All procedures were performed according to the Association for Research in Vision and Ophthalmology (ARVO) statement for the use of Animals in Ophthalmic and Vision Research. BALB/c mice of both sexes aged between 6 and 8 weeks were used in all experiments. They were obtained from Capital Medical University (Beijing, China). Mice were euthanatized by CO_2_ inhalation, followed by cervical dislocation.

### Cell culture medium and chemical reagents

Dulbecco’s modified Eagle’s medium/Ham’s nutrient mixture F12 (1:1 DMEM/F12) was purchased from Invitrogen (Carlsbad, CA), fetal bovine serum was purchased from Hyclone Laboratories (Logan, UT), insulin, transferrin, hydrocortisone, dimethyl sulfoxide, triton-X100, epidermal growth factor, and 4,6-diamidino-2-phenylindole were purchased from Sigma-Aldrich (St. Louis, MO). NGF (2.5S βNGF Grade I) was purchased from Millipore (Bedford, MA). Goat antimouse p63 and MUC5AC antibodies, fluorescein isothiocyanate-conjugated rabbit antigoat secondary antibody and rodamine-conjugated rabbit antigoat secondary antibody were from Santa Cruz Biotechnology (Santa Cruz, CA).

### Primary culture of mouse limbal epithelial cells

Immediately after the mice were euthanatized, the eyes were removed, and a 2 mm band of the superficial tissue from the limbus was excised. Then the tissue was placed into PBS containing 300 μg/ml penicillin-streptomycin. It was finely minced into 1 mm^3^ pieces and anchored onto 96-well culture dishes. The culture dishes contained just enough medium to cover the bottom of the dishes. The medium was a 1:1 mixture of DMEM/F12 supplemented with 5% fetal bovine serum, 100 μg/ml penicillin-streptomycin, 5 μg/ml insulin, 0.5 μg/ml hydrocortisone, 10 μg/ml transferrin, 10 ng/ml EGF, and 0.5% dimethyl sulfoxide. Cultures were incubated at 37 °C with 5% CO_2_. After 24 h, about 150 μl medium was added. Then the pieces were removed when the cells grew from the tissue and were surrounding them. The medium was changed every 3 d. NGF at the concentrations of 0 ng/ml (control group), 10 ng/ml, 100 ng/ml, and 250 ng/ml was added to the medium when the cells were at 80% confluence. The concentrations of NGF were chosen as described in Lambiase et al. [11]. Then the cells were cultured for 24 h to 5 d, respectively.

### Immunofluorescence staining for 63-kDa protein (p63) and mucin-5AC

For p63 staining, the cells were fixed with methanol for 15 min at about 80% confluence. After three washes with PBS for 3 min, the cells were incubated in PBS containing 0.1% triton-X100 for 15 min at room temperature, then preincubated with normal rabbit serum 10% for 15 min to block nonspecific staining. Cultures were incubated with goat antimouse p63 primary antibody (1:50) for 12 h at 4 °C. After three rinses with PBS for 5 min, the cultures were incubated with rodamine-conjugated rabbit antigoat secondary antibody (1:200) for 1 h at 37 °C, followed by three washes in PBS and nuclei counterstaining with 4,6-diamidino-2-phenylindole . Then cultures were visualized by fluorescence microscopy (Olympus, Tokyo, Japan). The number of p63-positive cells and total cells in ten randomly-selected fields were measured, and the positive rate was calculated.

For MUC5AC staining, the cells were fixed at different time points after they were grown in the medium containing NGF. Goat antimouse MUC5AC primary antibody (1:50) and fluorescein isothiocyanate-conjugated rabbit antigoat secondary antibody (1:200) were used and incubated with cells for 12 h at 4 °C and 2 h at room temperature, respectively. Finally, the percentage of MUC5AC-positive cells (goblet cells) was evaluated as described above.

### Real-time PCR

After each time point of treatment, RNA was extracted from the limbal epithelial cells according to the manufacturer’s instructions (Biomed, Beijing, China). Briefly, 1 ml trizol (Sigma-Aldrich, St. Louis, MO) were added to the cells sample, and then cells were treated with 200 μl chloroform. After certrifugation, supematant was removed. The extracted RNA pellet was washed with 70% ethanol and certifuged at 12,000 r/min for 45 s at 4 °C. At last, RNA was stored at -70 °C until it was used. The cDNA were then synthesized from 3 μl RNA template in a 25 μl reaction. The test gene primer and probe sets were optimized for concentration and amplification efficiency. An assay for the ubiquitous housekeeping gene glyceraldehyde-3-phosphate dehydrogenase (*GAPDH*) served as an endogenous reference. Real-time PCR was performed by using the Line-Gene Real Time Quantitative PCR System (BIOER technology, Hangzhou, China). The *MUC5AC*-specific primers were sense 5′-AAC CCG TGT GTG ACT CAT AA-3′, and antisense 5′-TCA TAG CAG CAT CCG TCT T-3′ (NM_010844). The PCR conditions were 95 °C for 2 min, followed by 45 amplification cycles on a real-time PCR system (95 °C for 20 s, 58 °C for 25 s, and 72 °C for 30 s). For each sample, analyses were performed in triplicate.

### Statistical analysis

Data were expressed as mean±SEM. SPSS 11.0 (SPSS Inc., Chicago, IL) was used for statistical comparison. The rates of MUC5AC-positive cells were compared using the SNK-q test. P values of >0.05 were considered statistically significant.

## Results

### Morphology of limbal epithelial cells and examination of limbal epithelial progenitor cells in vitro

**Figure 1:**
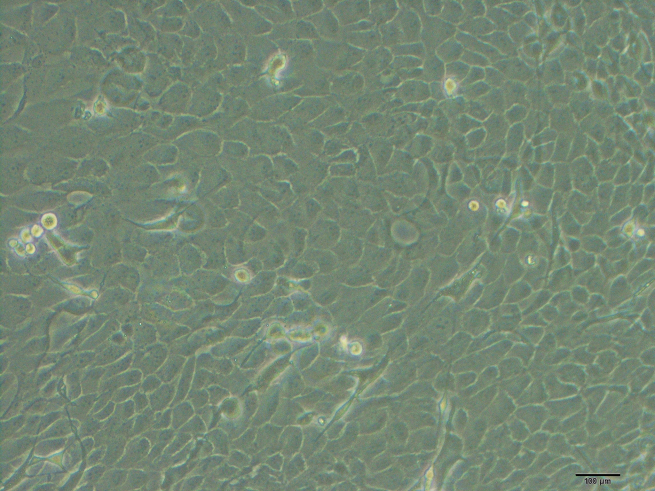
Characterization of  primary culture of mouse limbal epithelial cells. Phase contrast microscopy showed the morphology of limbal epithelial cells. Cells appeared to be compact, uniform and cobblestone pavement in shape.

As early as 24 h after seeding, cells were visible around the tissue. By day 6 or 7, the cells were at 80% confluence. The limbal epithelial cells morphology in primary culture appeared to be compact, uniform, and cobblestone pavement in shape (Figure 1).

**Figure 2:**
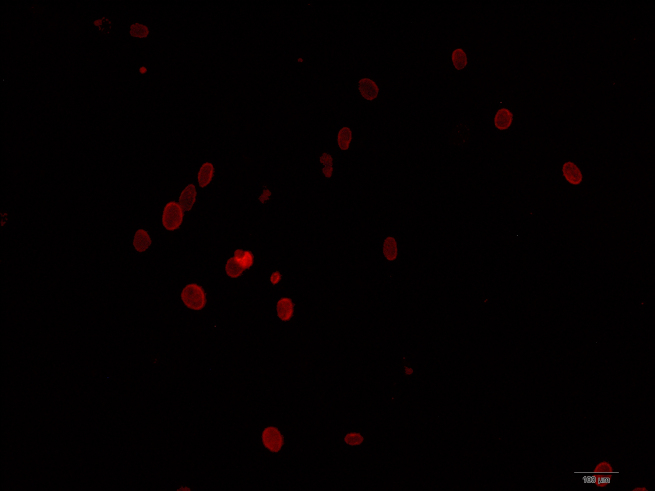
Immunofluorescence staining of 63-kDa protein in primary culture of mouse limbal epithelial cells. The 63-kDa protein positive cells (red) indicated the presence of the limbal epithelial progenitor cells.

Immunofluorescence staining revealed that some limbal epithelial cells were positive for p63 (Figure 2); this protein is indicative for predifferentiation status and suggests the presence of progenitor cells.

### Elevation of the expression of *mucin-5AC* mRNA

**Figure 3:**
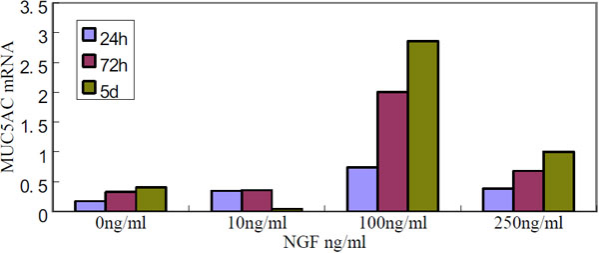
Nerve growth factor modulated the expression of *MUC5AC* mRNA. The results of Real-time PCR showed that the expression of *MUC5AC* mRNA was upregulated significantly after the limbal epithelial cells were cultured in the medium containing 100 ng/ml nerve growth factor, compared to the control group (0 ng/ml). The levels of *MUC5AC* mRNA were time-dependent with 100 ng/ml nerve growth factor.

To investigate the effect of NGF on mucin expression, *MUC5AC* mRNA was analyzed by real-time PCR. The results showed that the expressions of *MUC5AC* mRNA were upregulated significantly after the cells were cultured in the medium containing 100 ng/ml NGF, compared to the control group (Figure 3), and the levels were time-dependent. When the cells were treated with 250 ng/ml NGF, *MUC5AC* mRNA expression slightly increased.

### Nerve growth factor modulation of goblet cell differentiation

**Figure 4:**
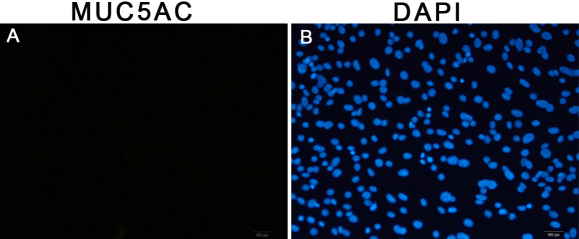
Immunofluorescence staining of mucin-5AC in the control group (0 ng/ml nerve growth factor) at the time point of 5 d. The results showed that mucin-5AC positive cells (goblet cells) were not detected when cultured for 5 d (**A**). Nuclei were stained with 4,6-diamidino-2-phenylindole (**B**; blue).

Immunofluorescence staining of MUC5AC was used to identify goblet cells. In the control group, no MUC5AC-positive cells were detected at each time point (Figure 4). After the cells were treated with 10 ng/ml NGF, immunofluorescence staining of MUC5AC was negative at each time point.

**Figure 5:**
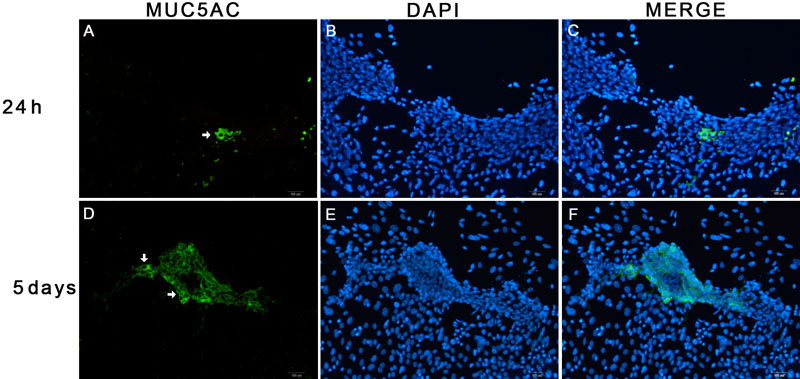
Immunofluorescence staining of mucin-5AC in the limbal epithelial cells treated with 100 ng/ml nerve growth factor. A few of goblet cells (green, white arrows) were detected at 24 h (**A**), the mumber of goblet cells increased at the time point of 5 d comparing with 24 h (**D**; p<0.05). Nuclei were stained with 4,6-diamidino-2-phenylindole (**B**, **E**; blue). Double immunolabeling for mucin-5AC and 4,6-diamidino-2-phenylindole indicated the presence of goblet cells in primary culture of mouse limbal epithelial cells with 100 ng/ml nerve growth factor (**C**, **F**).

When the cells were cultured in the medium containing 100 ng/ml NGF, MUC5AC-positive cells were detected at each time point of treatment. The ratios of positive cells were 5.27±6.15% at 24 h, 8.11±9.17% at 72 h, and 18.47±11.65% at 5 d, respectively. The number of MUC5AC-positive cells had significantly increased at 5 d, compared with those at 24 h and 72 h (p<0.05; Figure 5). However, there was no significant difference between 24 h and 72 h (p>0.05). When the cells were treated with 250 ng/ml NGF, there were only a few MUC5AC-positive cells after 5 d incubation. No positive cells were detected at other time points.

## Discussion

Although it has been reported that both goblet cells and conjunctival epithelial cells come from a bipotent conjunctival precursor cell [4,12], there is some evidence showed that they might be generated from corneal limbal stem cells. The study of epithelial cell streaming in rat conjunctiva showed that bulbar conjunctiva and palpebral conjunctiva were two independent cellular kinetic systems. Bulbar conjunctiva may originate in the corneal limbus, and corneal limbus stem cells could be generated into two epithelial cell lines, one corneal and the other conjunctival [13]. A recent study also indicated that corneal limbus contained oligopotent stem cells with the capacity to generate individual colonies of corneal and conjunctival cells as a transitional zone [5]. Consequently for this study, corneal limbal epithelial cells were chosen, and their capability to differentiate into goblet cells was observed.

NGF is a neurotrophic factor that can stimulate growth and differentiation in neurons [14]. It not only has the effect on cells of neuronal origin but also has a range of functions outside the nervous system, especially on the ocular surface. It was reported that NGF could promote corneal healing in vitro and in vivo [7] and could also play an important part in ocular allergies, for example, vernal keratoconjunctivitis [15].

It has been reported that NGF was associated with ocular mucins and goblet cells. Topical NGF treatment was indicated to induce an increase in goblet cell density in an animal model of dry eye [9]. A recent study demonstrated that NGF induced epithelial differentiation and dose-dependent increases both in the number of goblet cells and in *MUC5AC* mRNA expression in both the cell line and primary culture of the human conjunctival epithelium [15]. Our previous study showed that NGF could cause a dose-dependent increase in the proliferation of human goblet cells in vitro [16].

The effect of NGF on corneal limbal epithelial cells in vitro has already been studied. It has been reported that both the high affinity the NGF receptor TrkA and the low affinity of the receptor p75 are expressed by the basal cells of the human limbal epithelium [17-19]. Also reported was the TrkA signaling pathway’s important role in the expression of limbal epithelial cells on amniotic membranes in vitro [17]. Moreover, NGF was observed to stimulate the proliferation of limbal epithelial cells only at a high concentration (250 ng/ml) [6], but Kruse et al. [6] only investigated the effect of NGF on the differentiation of limbal epithelial cells into corneal epithelial cells. The results the present study demonstrate that NGF could promote the differentiation of corneal limbal epithelial cells to goblet cells and increase the expression of *MUC5AC* mRNA in a primary culture of mouse limbal epithelial cells. However, this effect was not dose-dependent. Further studies are needed to determine whether the effect of NGF on the differentiation of limbal epithelial cells into goblet cells is mediated by TrkA or p75.

In summary, NGF may promote the differentiation of corneal limbal progenitor cells into goblet cells and increase the expression of *MUC5AC* at a concentration of 100 ng/ml in a primary culture of mouse limbal epithelial cells. This NGF effect needs further study with animal models in vivo.
